# The Use of Catheter Mount Will Result in More Reliable Carbon Dioxide Monitoring under Fluid Exposing Conditions

**DOI:** 10.1155/2019/4120127

**Published:** 2019-07-01

**Authors:** Yongil Cho, Wonhee Kim, Tae Ho Lim, Hyuk Joong Choi, Jaehoon Oh, Bossng Kang, Youjin Kim, In Young Kim

**Affiliations:** ^1^Department of Emergency Medicine, College of Medicine, Hanyang University, Seoul, Republic of Korea; ^2^Department of Emergency Medicine, College of Medicine, Hallym University, Seoul, Republic of Korea; ^3^Department of Biomedical Engineering, College of Medicine, Hanyang University, Seoul, Republic of Korea; ^4^Department of Plant Biology, Rutgers, The State University of New Jersey, New Brunswick, New Jersey, USA

## Abstract

**Introduction:**

Capnometer can be readily malfunctioned by fluid exposure during treatment of critically ill patients. This study aimed to determine whether placing capnometer distant from the endotracheal tube by connecting direct connect catheter mount (DCCM) is effective in yielding reliable end-tidal carbon dioxide (ETCO_2_) by reducing capnometer malfunctioning caused by water exposure.

**Methods:**

In 25 healthy adults, a prospective, open label, crossover study was conducted to examine the effect of DCCM in mainstream and microstream capnometers under water exposing conditions. The primary endpoint was the comparison of ETCO_2_ between proximal DCCM (pDCCM) and distal DCCM (dDCCM).

**Results:**

For mainstream capnometers, mean ETCO_2_ was significantly (*p* < 0.001) higher in dDCCM compared to pDCCM under water exposing conditions (29.5 vs. 19.0 with 5 ml; 33.8 vs. 21.2 with 10 ml; mmHg). Likewise, for microstream capnometers, ETCO_2_ was greatly higher (*p* < 0.001) in dDCCM compared to pDCCM (30.5 vs. 13.9 with 5 ml; 29.9 vs.11.4 with 10 mL; mmHg). ETCO_2_ measured by dDCCM was reliable in microstream settings, whereas it was unreliable in mainstream (correlation coefficient 0.88 vs. 0.27).

**Conclusions:**

Application of DCCM onto the capnometer setting seems to be effective in reducing capnometer malfunctioning under fluid exposing conditions, which is obvious in microstream capnometer by producing more reliable ETCO_2_.

## 1. Introduction

Capnometer is a device that measures end-tidal carbon dioxide (ETCO_2_) by infrared sensor [1, 2]. Capnometer has been widely used to monitor proper placement of endotracheal tube (ETT) and status of ventilation [3, 4]. According to the 2010 American Heart Association (AHA) and European Resuscitation Council (ERC) guidelines, the use of capnometer is recommended during cardiopulmonary resuscitation (CPR), in order to evaluate quality of CPR and detect recovery of spontaneous circulation (ROSC) in intubated patients [5, 6]. During CPR, while low ETCO_2_ (<10 mmHg) represents poor quality of CPR, a dramatic increase of ETCO_2_ (up to 35-40 mmHg) indicates the occurrence of ROSC [6–8]. Accurate assessment of ETCO_2_ is therefore essential to monitor placement of ETT and quality of CPR.

Nevertheless, reliable ETCO_2_ measurement by capnometer is difficult in clinical settings. ETCO_2_ measurement becomes readily unreliable when capnometer is vulnerable to fluid exposure through ETT, mostly in cases of wet lung conditions [1, 4]. In wet lung conditions, fluid produced by patients is present in ETT, which interferes the infrared sensor on capnometer with detecting CO_2_ absorption at 4.3 *μ*m wavelength, resulting in under- or oversensing ETCO_2_ values. Furthermore, the sampling line connected to sidestream capnometer (i.e., microstream capnometer) is often occluded by condensed particles of fluid sourced from patients [1, 4]. Therefore, clinical settings of which patients have wet lung conditions often render capnometer exposed to fluid through ETT, leading to higher chances of capnometer malfunctioning.

To achieve accurate ETCO_2_ measurement, it is imperative for capnometer to avoid fluid contact and in that perspective, placing capnometer away from ETT is necessary. The direct connect catheter mount (DCCM, RT021 catheter mount, Fisher & Paykel Healthcare Ltd., Auckland, NZ) is a tubing system commonly inserted between breathing circuit and ETT to provide this connection with flexibility and a resultant reduction in extubation risk [9]. However, it can also be placed between ETT and capnometer to make them separate away, which could help capnometer avoid fluid contact supplied from patients.

The purpose of this study was to investigate whether placing capnometer away from ETT via DCCM insertion is effective in yielding reliable ETCO_2_ by protecting capnometer against water contact. We hypothesized that the use of DCCM will reduce capnometer malfunction, leading to reliable ETCO_2_ monitoring.

## 2. Materials and Methods

### 2.1. Study Design and Recruitment

A prospective crossover study was conducted at Hanyang University Medical Center in Seoul, Republic of Korea, on March 17, 2014. The study protocol was approved by the Institutional Review Board (IRB) of the Hanyang University Guri Hospital (Approval date: March 2014; Reference no. 2013-68). Recruitment was performed on March 9 - 16, 2014, and 25 healthy adults who submitted their written consent forms were included in the study. All patients were recruited after IRB approval and registration with clinicaltrials.gov.

### 2.2. Protocols

**Figure 1:**
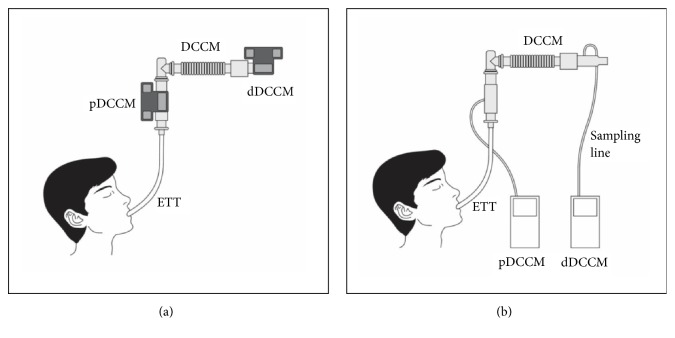
*Schematic figures of (a) mainstream capnometers and (b) microstream capnometers*. Abbreviations: DCCM, direct connect catheter mount; dDCCM, distal capnometer of direct connect catheter mount; ETT, endotracheal tube; pDCCM, proximal capnometer of direct connect catheter mount.

Two different sets of capnometers were tested in the study: mainstream capnometer vs. microstream capnometer. Two capnometers for each set were installed at proximal and distal ends of DCCM. For both settings, capnometers installed on proximal DCCM (pDCCM) were directly linked to ETTs, being free from the DCCM effect, whereas those on distal DCCM (dDCCM) were under the DCCM effect. In this study, hence, four test groups were designed: (1) mainstream-pDCCM; (2) mainstream-dDCCM; (3) microstream-pDCCM; and (4) microstream-dDCCM (Figure 1).

Prior to the study, all participants were provided with 5 min oral instructions on breathing techniques. During inspiration, participants were asked to inhale through nose only, not allowing them to open their mouth and swallow water. During expiration, however, they were forced to occlude their nose and exhale forcefully only through mouth when exposed to the water-sprayed ETT. Participants were also asked to breathe regularly according to the metronome sound (15 beats per min), which was made by the Micro Metronome application (SPACEWARE Inc., Android apps on Google Play) installed on the smart phone (LG Optimus G Pro, LG Electronics, Seoul, Republic of Korea).

**Figure 2:**
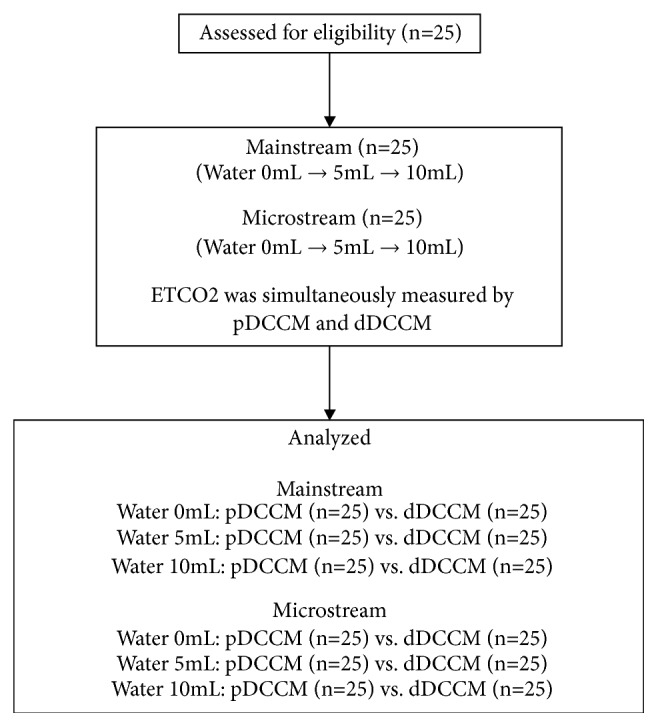
*Flow chart of experimental groups*. Abbreviations: dDCCM, distal capnometer of direct connect catheter mount; ETCO_2_, End-tidal carbon dioxide; pDCCM, proximal capnometer of direct connect catheter mount.

All participants (n=25) used one of the mainstream and microstream capnometers and then the other capnometer. They were instructed to hold the ETT cuffs by their mouth and breathe for 2 min each time when the ETT was sprayed with 0 (baseline), 5, or 10 ml distilled water, in turn, which were given to the ETT just before connecting with pDCCM. Five min intervals were given for each time of breathing. The water sprayed within the ETT was allowed to contact capnometers freely when participants were exhaling. During crossovers, 30 min intervals were given to all participants. All ETCO_2_ values measured after each time of breathing were recorded from different capnometers. All capnometers were calibrated regularly each time before initiating breathing tests of individual participant. The flow sampling rate in microstream capnometer was 50 ml/min. Sampling lines and adapters installed on capnometers were also changed accordingly (Figure 2).

The primary endpoint is the comparison of ETCO_2_ values measured in water exposing conditions by pDCCM and dDCCM in both mainstream and microstream settings. The secondary endpoint is the reliability of ETCO_2_ values measured by dDCCM among three different water conditions in both mainstream and microstream settings.

### 2.3. Equipment

Two types of portable capnometers were used in this study: (1) EMMA™ Mainstream Capnometer (Masimo Corp., CA, US) equipped on to EMMA airway adapter, which operates at -5 to 50°C temperature with 10-95% humidity [10]; and (2) Microcap® Plus Microstream Capnography (Oridion Medical Ltd., Jerusalem, Israel) connected to Smart CapnoLine Plus sampling line (Oridion Medical Ltd., Jerusalem, Israel) [11]. By detecting infrared absorption, both capnometers can measure ETCO_2_ within the range of 0-99 mmHg. Direct connect catheter mount (DCCM) is at the length of ~15 cm with mechanical dead space equivalent to 25 ml. The ETT (Mallinckrodt™ Hi-Lo Oral/Nasal Tracheal Tube Cuffed Murphy Eye, Covidien, MA, USA) utilized was 7.5 size with 15 ml mechanical dead space [12].

### 2.4. Statistical Analysis

Prior to the experiments, minimum sample size was calculated based on our pilot study results. Briefly, when ETT was sprayed with 5 ml water, mean ETCO_2_ values measured by pDCCM and dDCCM were 14.4 ± 19.9 mmHg and 29.2 ± 7.4 mmHg, respectively. Difference from dDCCM to pDCCM was 14.7 ± 20.6 mmHg. Sample size was calculated by Wilcoxon-signed rank test using G-power software (version 3.1.7; Heine Heinrich University, Düsseldorf, Germany). By using *α* = 0.05 and *β* = 0.05, it was concluded that at least 21 participants are required.

SPSS software (version 20; IBM Corp, NY, USA) was used for data analysis. All variables were analyzed with Shapiro-Wilk normality test. ETCO_2_ values measured in water sprayed conditions were analyzed by repeated measures ANOVA. The graphs showing individual ETCO_2_ values monitored from every breath over time were represented as mean with standard errors (95% CI) using error bars. We analyzed the agreement of ETCO2 measurement between pDCCM and dDCCM using Bland-Altman plots. We also compared the agreement of ETCO2 measurement between mainstream and microstream capnometer. The intraclass correlation coefficient (ICC) was calculated for ETCO_2_ values of dDCCM to estimate interrater reliability among three different water conditions. All data are shown as means ± SD.* p*-value < 0.05 was considered significant.

## 3. Results

### 3.1. Overall ETCO_2_ Measured by pDCCM and dDCCM

Table 1 shows the overall characteristics of the 25 participants, and Table 2 shows overall ETCO_2_ measured by pDCCM and dDCCM. For both mainstream and microstream capnometers, ETCO_2_ values measured by pDCCM and dDCCM were compared under three different water conditions (0, 5, or 10 ml water sprays) (Table 2). For mainstream capnometers, ETCO_2_ measurements at baseline did not show statistically significant differences (*p *= 0.09) between pDCCM (34.5 ± 6.5 mmHg) and dDCCM (31.9± 4.9 mmHg). However, when 5 and/or 10 ml water sprays were given to the ETT, ETCO_2_ measurement was significantly (*p *< 0.001) higher in dDCCM than in pDCCM (29.5 ± 7.0 mmHg vs. 19.0 ± 23.5 mmHg with 5 ml water; 33.8 ± 14.8 mmHg vs. 21.2 ± 24.5 mmHg for 10 ml water). Likewise, for microstream capnometers, ETCO_2_ measurements at a baseline were similar (*p *= 0.83) between pDCCM (32.5 ± 3.7 mmHg) and dDCCM (32.7 ± 4.3 mmHg), whereas with 5 and/or 10 ml water sprays, ETCO_2_ measurement was greatly (*p *< 0.001) higher in dDCCM than in pDCCM (30.5 ± 5.1 mmHg vs. 13.9 ± 15.2 mmHg with 5 ml water; 29.9 ± 4.3 mmHg vs.11.4 ± 14.4 mmHg with 10 mL water). These results indicate that the use of DCCM can reduce capnometer malfunctioning under water exposing conditions.

### 3.2. Individual ETCO_2_ for Each Breath Measured by pDCCM and dDCCM

**Figure 3:**
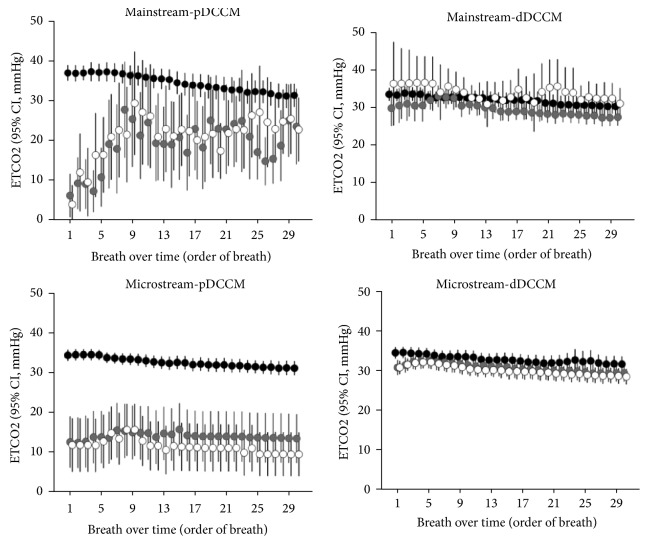
*Individual ETCO*_*2*_ *for each breath over time measured by pDCCM and dDCCM under different water conditions (black circle - no water, grey circle - 5 ml water, white circle - 10 ml water) created within the endotracheal tube*. Individual ETCO2 values monitored from every breath over time were represented as mean with standard errors (95% CI) using error bars. Abbreviations: dDCCM, distal capnometer of direct connect catheter mount; ETCO_2_, End-tidal carbon dioxide; pDCCM, proximal capnometer of direct connect catheter mount.

Individual ETCO_2_ values for each breath over time measured by pDCCM and dDCCM are shown in Figure 3. For both mainstream and microstream capnometers when treated with 5 and/or 10 ml water sprays, ETCO_2_ measurements by pDCCM were far lesser than those at baseline (0 ml water), whereas these measurements by dDCCM were very close to those at baseline. In the Bland-Altman plot, wider range of the 95% limits of agreement was shown in the water exposing conditions (5, 10mL in ETT) comparing with baseline (0mL in ETT) for both capnometers, which suggests inaccuracy of pDCCM under water exposing conditions [13]. Additionally, the microstream capnometer showed better agreement between pDCCM and dDCCM than mainstream capnometer (Supplementatary Figure S1).

### 3.3. Reliability of ETCO_2_ Measured by dDCCM

For both mainstream and microstream capnometers, the reliability of ETCO_2_ measured by dDCCM was assessed among three different water conditions (0, 5, or 10 ml water sprays). When using mainstream settings, ETCO_2_ measured by dDCCM was unreliable (average measure ICC 0.27, 95% CI 0.17-0.35;* p *< 0.001). On the contrary, ETCO_2_ measured by dDCCM was reliable in microstream settings (average measure ICC 0.88, 95% CI 0.87-0.89;* p *< 0.001) among three different water conditions. In the analysis using Bland-Altman plot, dDCCM showed better agreement between microstream and mainstream capnometer than pDCCM (Supplementatary Figure S2).

## 4. Discussion

This is a concept study that simulates fluid using water and evaluates the efficacy of DCCM in yielding reliable ETCO_2_ under fluid exposing conditions. The key finding from this study is that adoption of DCCM can reduce capnometer malfunctioning particularly when fluid inflow occurs through the ETT. It was evident from the results that, under water exposing conditions (5 or 10 ml water sprays), ETCO_2_ measurements were significantly lower in pDCCM than in dDCCM in both mainstream and microstream capnometers. With water, additionally, all ETCO_2_ values measured by pDCCM were far less than baseline ETCO_2_ (0 ml water spray), indicating that these levels of water caused pDCCM malfunctioning. On the contrary, ETCO_2_ values obtained from dDCCM placing away from water sources were very similar to the baseline ETCO_2_, implying that securing some distance from patients (~15 cm) [9] by DCCM installation seems to be effective in protecting against capnometer malfunctioning by lessening water vapor concentrations and inhibiting the capnometers from direct water contact.

We did not set partial pressure of carbon dioxide (PaCO_2_) in arterial blood gas analysis as a reference standard. Since there could be wide gap between ETCO_2_ and PaCO_2_ under water exposing conditions, baseline ETCO_2_ (0 ml water spray) was set to the reference standard.

ETCO_2_ monitoring is most reliable and accurate method to monitor success for tracheal intubation or CPR quality. However, under fluid exposing conditions, fluid could hinder obtaining reliable ETCO_2_ in proximal ETT. Additionally, in cases of patients receiving CPR or having fluid, low ETCO_2_ could be frequently observed. This study suggests that the use of DCCM could provide benefit to obtain more reliable ETCO_2_ by using DCCM in those cases.

It is of interest that, under water exposing conditions, the use of DCCM was more effective in microstream capnometer than in mainstream capnometer. It could be explained by the fact that the infrared sensor of mainstream capnometer was directly exposed to water, being vulnerable to being malfunctioning, whereas that of microstream capnometer was indirectly exposed to water via sampling lines connected to the opposite direction of gravity, implying that a continuous measurement of ETCO_2_ is possible unless there is water condensation and/or direct water contact.

In this study, two types (mainstream vs. microstream) of capnometers were used for CO_2_ monitoring. Depending on the use of gas sampling system, capnometers can be classified into two categories such as mainstream capnometer and sidestream capnography [1, 14], and the microstream capnograph used in this study is a type of sidestream capnography [14]. Mainstream capnometer can measure ETCO_2_ directly by using infrared sensor and does not need gas sampling system [1, 3]. Both the sidestream and microstream capnographs can monitor CO_2_ aspirated by sampling line [1, 3]. Water vapor can cause condensation in sample lines which can thus interfere with CO_2_ monitoring [1]. The occurrence of capnometer malfunctioning caused by water condensation is more often in sidestream capnograph compared to microstream capnograph. It is also known that, compared to mainstream capnometer, sidestream capnograph is more vulnerable to water itself [3]. It is the reason why mainstream capnometer and microstream capnograph were chosen for this study in assessing the level of capnometer malfunctioning under water exposing conditions.

This study was performed under water exposing conditions, where two different water conditions were simulated by spraying 5 ml or 10 ml of distilled water into ETT. These water amounts were equivalent to one-third (5 ml) or two-thirds (10 ml) of mechanical dead space of tracheal tube (15 ml), which was gauged by filling water into the tracheal tube.

From the study, we found that, in water exposing conditions, measuring ETCO_2_ is more accurate when using DCCM. In spite of it, using dDCCM to monitor ETCO_2_ is not the way traditionally recommended. Earlier studies indicated that ETCO_2_ measurement should be performed at the proximal point of tracheal tube (pDCCM) connected to the ventilator circuit [15], in order to avoid possible inconsistency between PaCO_2_ and ETCO_2_ when measured by dDCCM. We found that a baseline ETCO_2_ value for mainstream capnometers was significantly lower in dDCCM than in pDCCM. However, when measured by microstream, mean ETCO_2_ values were not significantly different between pDCCM and dDCCM. We assume that it is originated from the difference of gas sampling technique in capnometer. A microstream capnometer withdraws a continuous sample of gas through a capillary tube from the patient's airway to the monitor and a water trap removes particles of water before measurement takes place. Hence, dDCCM of mainstream capnometer is more vulnerable to water vapor than microstream.

This study has several limitations. Firstly, use of DCCM might not guarantee the complete inhibition of capnometer malfunctioning during water exposing conditions, as installation of DCCM itself is not sufficient to block water contact completely, but rather it can help in reducing the capnometer malfunctioning by allowing a certain distance from the water sources. Secondly, in simulating fluid, pure water was used in this study to prevent ethical conflicts in healthy volunteers. The potential effect of other components of fluid on the infrared sensor of capnometer remains still unknown. Thirdly, when conducting this study, other resuscitation techniques (i.e., suctioning and bag valve mask ventilation) were excluded, as they can act as confounding factors when analyzing the sole effect of DCCM on capnometer malfunctioning. Fourthly, the CO_2_ rebreathing effect possibly occurring during DCCM mounting onto the ETT was incompletely corrected. As 25 ml dead space of DCCM can increase the CO_2_ rebreathing effect and elevate baseline ETCO_2_ for both pDCCM and dDCCM, a possible gap still exists between ETCO_2_ measurements obtained from this study and real CO_2_ levels from exhales of the participants. However, to avoid rebreathing effect, participants were guided to exhale forcefully to reach maximal flow rate and tidal volume.

## 5. Conclusions

In conclusion, application of DCCM onto the capnometer setting seems to be effective in reducing capnometer malfunctioning under fluid exposing conditions, which is obvious in microstream capnometer by producing more reliable ETCO_2_. To demonstrate the efficacy of DCCM in real world patients under fluid exposing conditions, further clinical studies are needed.

## Figures and Tables

**Table 1:** General characteristics.

Characteristics	Data
	(n=25)
Age (yr)	30.6 ± 4.2
Male sex	19 (76)
Height (cm)	170.7 ± 7.4
Weight (kg)	69.5 ± 11.9
*∗*IBW (kg)	65.5 ± 8.4
†BSA (m^2^)	1.8 ± 0.1
‡Predicted tidal volume (ml·kg^−1^)	458.8 ± 58.3
BMI (kg·m^−2^)	23.6 ± 2.6
Underlying lung disease	None
Carbohydrate beverage ingestion before study	None

Categorical variables are given as numbers (percentage). Continuous variables are given as mean ± SD.

*∗* Calculated by Devine formula; IBW (male) = 50 + 2.3 x (height over 60 inches); IBW (female) = 45.5 + 2.3 x (height over 60 inches)

† Calculated by Mosteller formula; BSA (m^2^) = (Height (cm) x Weight (kg) / 3600)^1/2^

‡ Calculated by the formula for tidal volume in health young adults; 7 (ml) x IBW (kg)

Abbreviations: BMI, body mass index; BSA, body surface area; IBW, ideal body weight; SD, standard deviation

**Table 2:** ETCO_2_ by using pDCCM and dDCCM.

Capnometer	Water sprays	*∗*ETCO_2_ (mmHg)			
		†pDCCM	‡dDCCM	Mean difference	§*p*-value
		(n=25)	(n=25)		
Mainstream	0 ml	34.5 ± 6.5	31.9 ± 4.9	-2.5 ± 5.2	0.09
	5 ml	19.0 ± 23.5	29.5 ± 7.0	10.4 ± 24.1	<0.001
	10 ml	21.2 ± 24.5	33.8 ± 14.8	12.5 ± 28.4	<0.001

Microstream	0 ml	32.5 ± 3.7	32.7 ± 4.3	0.2 ± 2.5	0.83
	5 ml	13.9 ± 15.2	30.5 ± 5.1	16.6 ± 16.7	<0.001
	10 ml	11.4 ± 14.4	29.9 ± 4.3	18.5 ± 16.0	<0.001

*∗* Values are given as mean ± SD.

†pDCCM, proximal capnometer of direct connect catheter mount

‡dDCCM, distal capnometer of direct connect catheter mount

§Calculated by repeated measures ANOVA.

Abbreviations: dDCCM, distal capnometer of direct connect catheter mount; ETCO_2_, End-tidal carbon dioxide; pDCCM, proximal capnometer of direct connect catheter mount; SD, standard deviation

## Data Availability

All data and materials in this study are fully available without restriction.

## Supplementary Materials

Supplementary Materials Figure S1. Bland-Altman plots for evaluating agreement of ETCO2 measurement between pDCCM and dDCCM under water exposing conditions (baseline, 5 mL water, 10 mL water). Figure S2. Bland-Altman plots for evaluating agreement of ETCO2 measurement between mainstream and microstream capnometer under water exposing conditions (baseline, 5 mL water, and 10 mL water).
