# Supplementary material for: The Use of Catheter Mount Will Result in More Reliable Carbon Dioxide Monitoring under Fluid Exposing Conditions
Source: Emerg Med Int. 2019 Jul 1;2019:4120127. doi: 10.1155/2019/4120127 (PMC6632495; doi:10.1155/2019/4120127)

**Supplementary Materials**

**Figure S1. Bland-Altman plots for evaluating agreement of ETCO_2_ measurement between pDCCM and dDCCM under water exposing conditions (baseline, 5 mL water, 10 mL water).**

Abbreviations: dDCCM, distal capnometer of direct connect catheter mount; ETCO_2_, End-tidal carbon dioxide; pDCCM, proximal capnometer of direct connect catheter mount


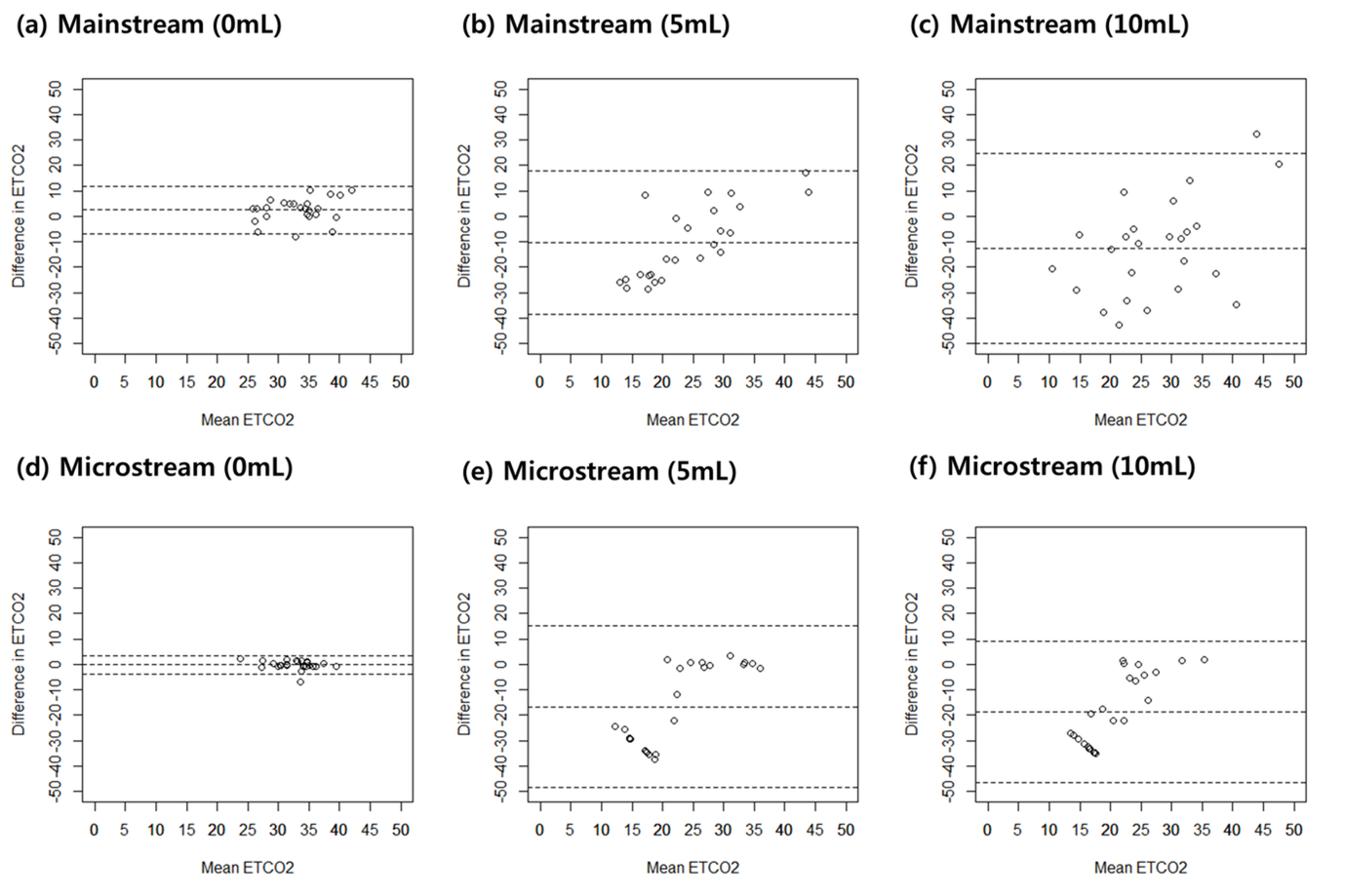


**Figure S2. Bland-Altman plots for evaluating agreement of ETCO_2_ measurement between mainstream and microstream capnometer under water exposing conditions (baseline, 5 mL water, 10 mL water).**

Abbreviations: dDCCM, distal capnometer of direct connect catheter mount; ETCO_2_, End-tidal carbon dioxide; pDCCM, proximal capnometer of direct connect catheter mount


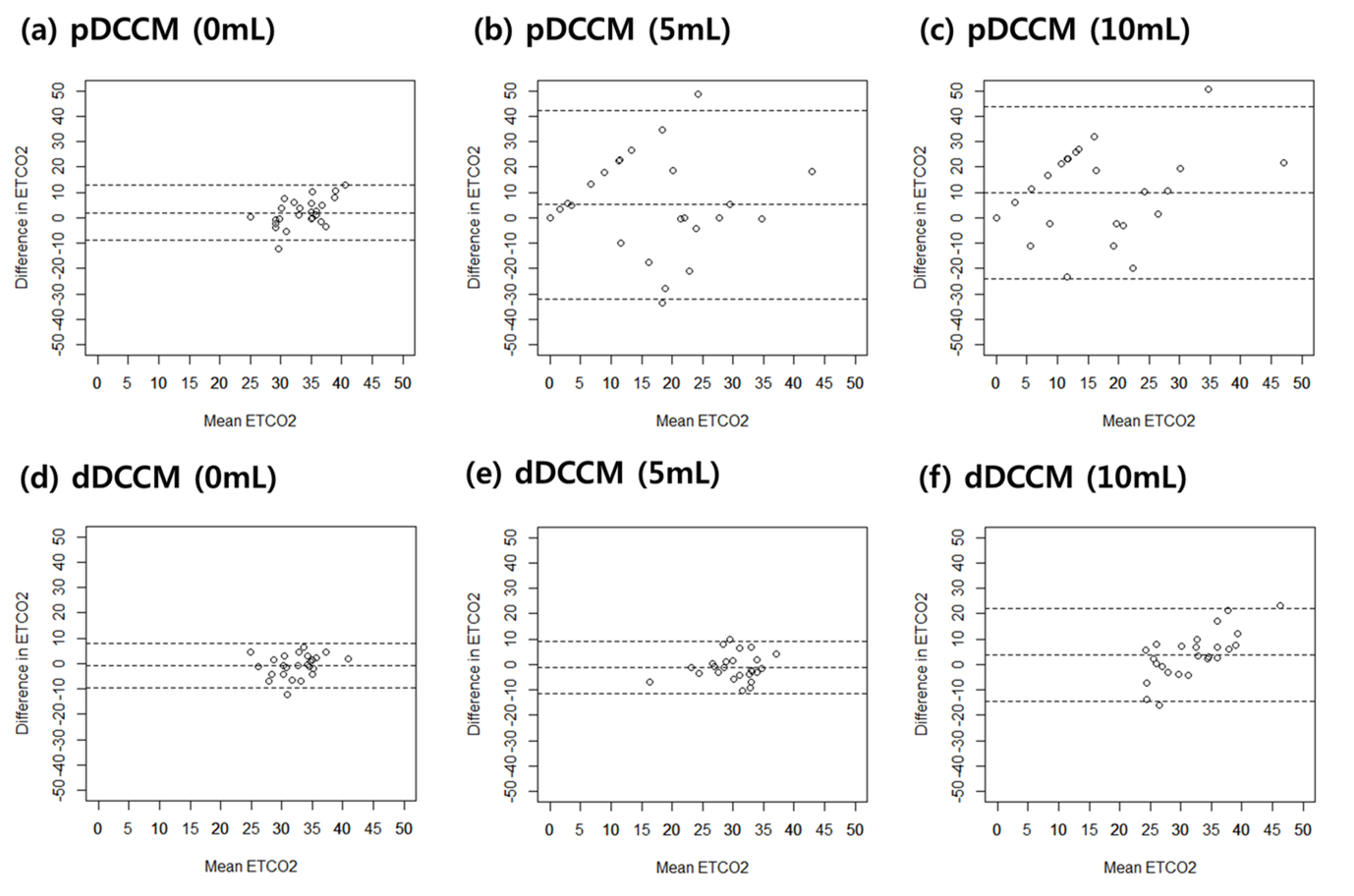

Supplement: Supplementary Materials — Figure S1. Bland-Altman plots for evaluating agreement of ETCO2 measurement between pDCCM and dDCCM under water exposing conditions (baseline, 5 mL water, 10 mL water). Figure S2. Bland-Altman plots for evaluating agreement of ETCO2 measurement between mainstream and microstream capnometer under water exposing conditions (baseline, 5 mL water, and 10 mL water). [file 4120127.f1.docx]
